# Diuretic Activity of a Novel Peripherally-Restricted Orally-Active Kappa Opioid Receptor Agonist

**DOI:** 10.3390/medsci7090093

**Published:** 2019-08-31

**Authors:** Tyler C. Beck, Matthew A. Hapstack, Gautam S. Ghatnekar, Thomas A. Dix

**Affiliations:** 1 Drug Discovery & Biomedical Sciences, Medical University of South Carolina, 280 Calhoun Street, QF204, Charleston, SC 29424-2303, USA; 2 College of Medicine, Medical University of South Carolina, 173 Ashley Ave., Charleston, SC 29424-2303, USA; 3 JT Pharmaceuticals, Inc., 300 West Coleman Blvd., Suite 203, Mount Pleasant, SC 29464-2303, USA

**Keywords:** diuretic, kappa, opioid, agonist, cardiovascular disease, heart failure

## Abstract

*Kappa*-opioid agonists (KOAs) enhance cardiac performance, as well as reduce infarct size and prevent deleterious cardiac remodeling following myocardial infarction. Additionally, KOAs promote diuresis; however, there has been limited development of KOAs as a class due to the promotion of untoward central nervous system (CNS)-mediated side effects. Our laboratory has developed a peripherally-restricted, orally-active, KOA (**JT09**) for the treatment of pain and cardiovascular disease. Peripherally-restricted KOAs possess a limited side-effect profile and demonstrate potential in preventing heart failure. The aim of this study was to assess the diuretic activity of lead compound **JT09** relative to vehicle control and Tolvaptan through single oral administration to adult male Sprague–Dawley rats. **JT09**-administered rats demonstrated significantly increased urine output relative to vehicle control. However, the effect persisted for 8 h, whereas Tolvaptan-administered rats demonstrated diuretic activity for 24 h. Relative to Tolvaptan, urine output was significantly reduced in **JT09** administered animals at all-time points, suggesting that the overall diuretic effect of **JT09** is less profound than Tolvaptan. Additionally, **JT09**-administered rats demonstrated alterations in clinical chemistry; reduced urine specific gravity; and increased urine pH relative to vehicle control. The following study establishes a preliminary diuretic profile for **JT09**.

## 1. Introduction

Cardiovascular disease (CVD) is the leading cause of death in the United States, accounting for over 800,000 deaths per year [1]. CVD costs society an estimated $351.2 billion per year in direct medical costs and indirect costs, such as lost wages. Furthermore, 6.2 million Americans suffered from heart failure (HF) between the years 2013 to 2016. Despite recent advances in cardiovascular therapies, the prevalence of HF continues to rise. 

Treatment for HF includes lifestyle changes and combination therapy with a variety of medications. Most common medications of use include β-adrenergic receptor antagonists (β-blockers), which reduce blood pressure, as well as reduce cardiac inotropy (contractility) and chronotropy (heart rate) [2]; Angiotensin converting enzyme (ACE)-inhibitors, which block the conversion of angiotensin I to angiotensin II, thus reducing blood pressure and mitigating deleterious cardiovascular remodeling; Angiotensin receptor antagonists, which act similar to ACE-inhibitors by blocking the activity of angiotensin II; and diuretics, which help relieve the symptoms of congestive heart failure by reducing volume overload and thus, reducing left ventricular pre-load and systemic vascular resistance [3]. There are several different classes of diuretics, including loop diuretics, thiazide diuretics, vasopressin receptor type 2 (V2) antagonists, and potassium-sparing diuretics [4,5,6]. Diuretics are commonly used together in tandem and are highly effective in providing symptomatic relief to patients with HF [7].

Despite the array of clinically-available medications for the treatment of HF, a single therapeutic that possesses diuretic activity, while also modulating cardiac neuro-hormonal activity and enhancing cardiac performance would be of significant value. Kappa-opioid agonists (KOAs) may serve as such agents. In addition to potent analgesic activity, KOAs have demonstrated cardiovascular, anti-pruritic, anti-edematous, anti-inflammatory, and anti-tussive effects [8]. KOAs have demonstrated the capacity to reduce infarct size following myocardial infarction when administered during reperfusion [9,10]. Additionally, KOAs reduce cardiac inotropy and chronotropy by inhibiting ß-adrenergic signaling, thus decreasing the symptoms and cardiac remodeling associated with HF [11,12,13]. Lastly, KOAs demonstrate potent diuretic activity, serving useful in restoring euvolemia in patients with heart and liver failure [14]. Despite their diverse activity, there has been limited development of KOAs as therapeutics due to the agonism of centrally-located kappa-opioid receptors (KORs), which leads to the promotion of untoward side-effects, including sedation, dysphoria, hallucinations, and psychosis [15,16,17,18]. Thus, the development of an orally-available, peripherally-restricted, KOA with a limited side-effect profile could serve as a broad spectrum therapeutic in the treatment of CVD.

**Figure 1:**
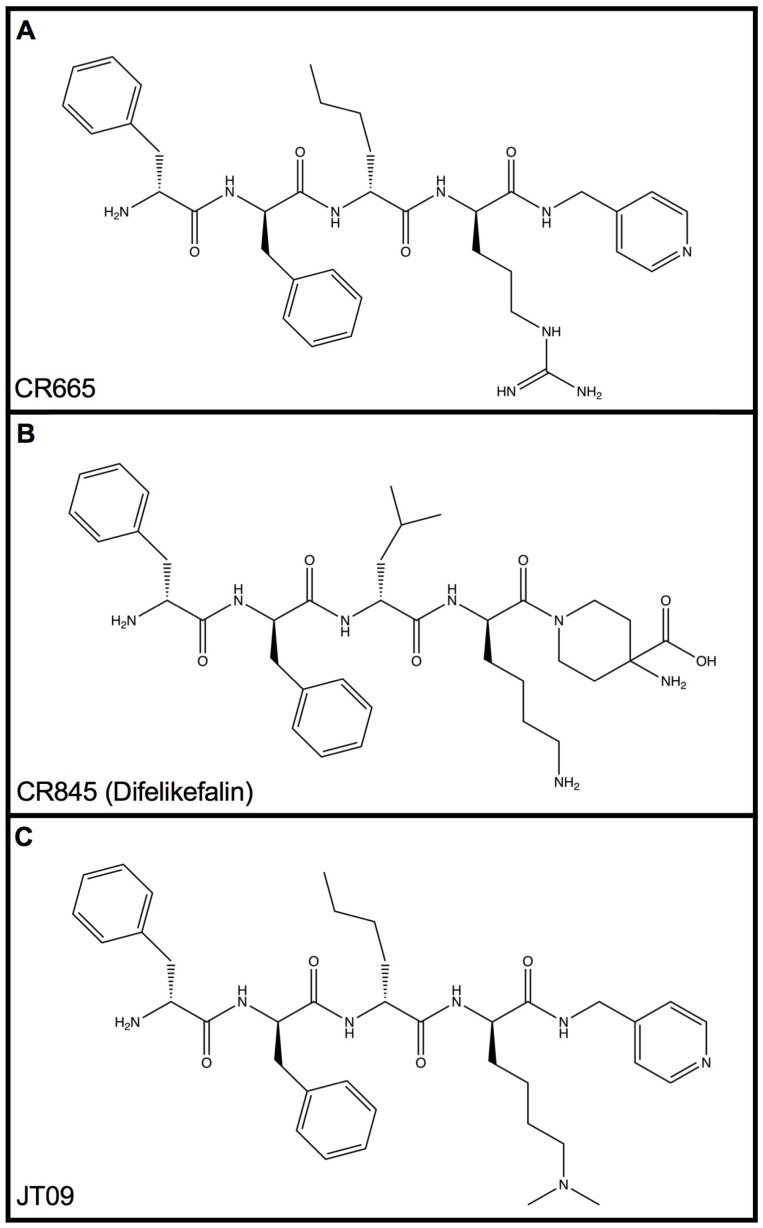
Chemical structures of CR665 (**A**), CR845 (**B**), and JT09 (**C**). The Position 4 D-Arg residue of CR665 was converted to a derivative containing a modified D-Lys residue in JT09.

The peptidic KOAs CR665 and CR845 (Figure 1), under development by Cara Therapeutics (Shelton, CT), do not cross the blood brain barrier (BBB) and have demonstrated potent analgesic, anti-inflammatory, and anti-edematous activity, without the negative side-effects associated with centrally-acting KOAs [19]. However, CR665 and CR845 do not have significant oral bio-availability, limiting their potential use in an out-patient setting. Our laboratory has developed proprietary modifications to the cationic amino acids arginine (Arg) and lysine (Lys) that impart oral bio-availability and improved stability in biological fluids when incorporated into peptides [20,21]. Earlier studies indicated that replacing the residue 4 D-Arg with modified D-Lys derivatives significantly improves pharmacokinetic/pharmacodynamics (PK/PD) characteristics [20,21,22,23]. Application of our laboratory’s peptide modification technology to the peripherally-restricted KOA CR665 generated derivatives demonstrating significantly improved oral bioavailability and PK/PD properties, while retaining the efficacy of the parent compound [24]. In a previous pain study, JT09 (Figure 1) demonstrated indistinguishable analgesic activity relative to morphine, while failing to promote the negative side-effects associated with centrally-acting opioid compounds. In this study, we screened for diuretic activity and clinical chemistry in rats receiving a single treatment of JT09 at various dose levels relative to controls. 

## 2. Materials and Methods 

### 2.1. Synthesis of Lead Compound

JT09 was assembled using standard Merrifield chemistry to build the peptide, while incorporating the non-natural D-Lys residues, synthesized as described previously [20,24,25,26,27,28,29,30]. All compounds were HPLC-purified and characterized by MALDI-mass spectrometry, giving measured [M+1]+ molecular weights within 0.1% of theoretical values.

### 2.2. Dose Selection and Justification

The dose of JT09 administered was selected using the oral (PO) ED_50_ and ED_90_ in the acetic acid induced rat writhing assay in previous studies [20,24]. 

### 2.3. Animals

All animal work was reviewed and approved by the Institutional Animal Care and Use Committee (IACUC) at the Medical University of South Carolina (IACUC-2018-00447). All experimental protocols were performed in accordance with the guidelines set forth in the NIH Guide for the Care and Use of Laboratory Animals, published by the Public Health Service. All protocols were performed on male Sprague–Dawley rats (Harlan, Prattville, AL, USA, 240–280 g), which were housed in an Association for Assessment and Accreditation of Laboratory Animal Care (AAALAC)-approved colony room maintained at a constant temperature and humidity. Furthermore, we exclusively used male rats in our preliminary assessment of the diuretic activity of JT09 in order to reduce the number of experimental animals studied. The literature suggests that there are no sex differences in the diuretic activity of KOAs when comparing male and female rats of equal weight [31]. A total of 20 animals (two per cage; approximately 175–225 g each) were kept on a 12-h light:dark cycle with ad libitum access to food and water. 

### 2.4. Diuretic Activity Study

Diuretic activity has been previously reported following administration of KOAs, especially at high dose levels. In this study, selected animals were administered either vehicle, Tolvaptan, or lead compound JT09 with urinalysis and serum chemistry performed. Required quantities of JT09 and Tolvaptan were dissolved in sterile phosphate buffered saline (PBS) for oral administration. Tolvaptan is a vasopression receptor type 2 (V2) antagonist that is used clinically to ameliorate the symptoms of congestive heart failure and serves as an acceptable positive control due to its oral bioavailability in rats and potent diuretic activity [4,6]. One hour prior to test item administration, rats were orally hydrated with reverse-osmosis purified water (25 mL/kg, PO) to ensure equal hydration status. For oral administration, vehicle (V) (0.2 mL, PO) and Tolvaptan (T) (30 mg/kg, PO) formulations were administered to negative and positive control animals, respectively. Similarly, J-5 and J-20 rats were orally-gavaged with 5 mg/kg and 20 mg/kg of JT09, respectively. Water bottles were removed before test item administration.

#### 2.4.1. Urinalysis

Following treatment, animals were housed individually in Techniplast metabolic cages (Braintree Scientific, Inc., Braintree, MA, USA) for urine collection, with samples taken at 30 min, 1, 2, 5, 8 and 24-h. The urine was analyzed based on the following parameters: appearance and color, volume (mL), pH, sodium concentration, and specific gravity. Analyses were performed using an Accutest 500 Urine Analyzer (Janal Pharmacol, Encino, CA, USA).

#### 2.4.2. Serum Chemistry

Blood was collected from all animals at 1, 5, and 24 h following drug administration. Isofluroane was used to establish mild anesthesia for blood collection (120 μL per sample) into gel-barrier tubes through retro-orbital plexus puncture, and serum was separated 5000 rpm for 10 min and analyzed for the parameters displayed in Table A1. A Comprehensive Diagnostic Profile was performed using a VetScan VS2 (Abaxis, Union City, CA, USA). Sodium (mmol/L), potassium (mmol/L), and chloride (mmol/L) were estimated using Prolyte Na/K/Cl analyzer (Diamond Diagnostics, Holliston, MA, USA). 

### 2.5. Statistics

Power calculations were performed to determine group size for all experiments described above. A maximum acceptable type I error of 5%, and an 80% probability for detection of a true difference between control and experimental groups for each condition described are necessary. Similar experiments reported in the literature by other investigators have resulted in a standard deviation of 25%. A difference of 30 to 35% will be considered significant. Therefore, the calculated sample size per group is five rats per cohort.

The data were verified and then subjected to statistical analysis using GraphPad Prism (GraphPad Software, Inc., La Jolla, CA, USA). Two-way mixed analysis of variance (ANOVA), followed by Dunnett’s post hoc test, was performed for different treatment and respective control groups. All analysis and comparisons were evaluated at the 95% level of confidence (P < 0.05). Statistically significant changes obtained from the aforementioned tests were designated by the superscripts throughout the report as stated below: statistically significant (* *p* < 0.05; ** *p* < 0.01; *** *p* < 0.001). Note: some data points are missing error bars because the error bars are shorter than the size of the symbol.

### 2.6. Data Compilation

Individual animal data are publicly available via figshare [32].

## 3. Results

### 3.1. Morbidity/Mortality and Clinical Signs

All animals of the vehicle, positive control, and test item groups were observed and appeared to be normal throughout the experimental period. No signs of clinical morbidity or mortality were observed throughout the duration of this study. 

### 3.2. Clinical Pathology

#### 3.2.1. Urinalysis

**Figure 2:**
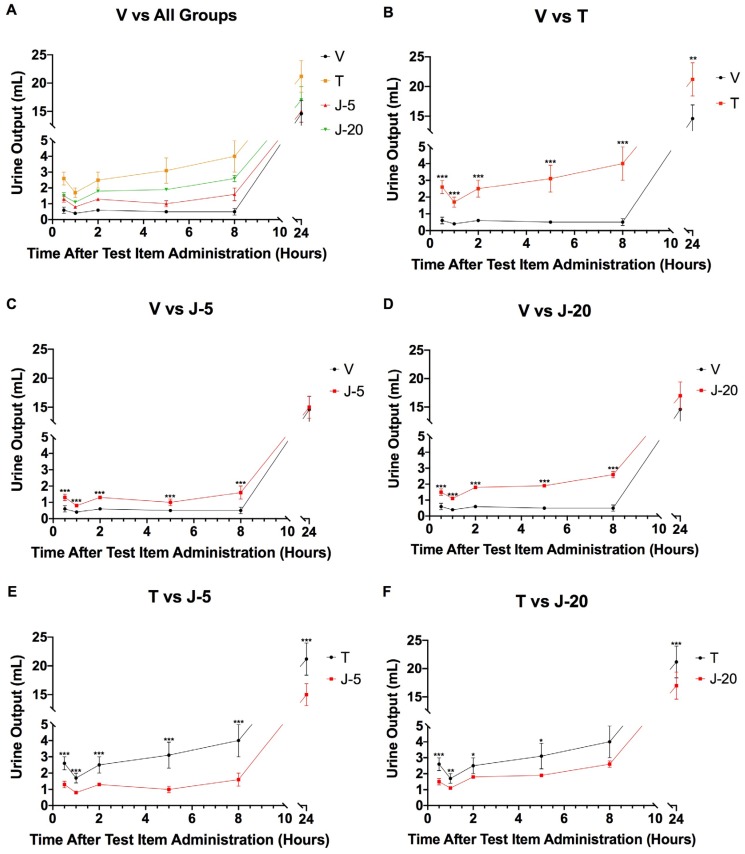
Average urine output measured at 30 min and 1 h, 2 h, 5 h, 8 h and 24 h after single oral or intravenous treatment. Figure (**A**) compares vehicle to all groups; (**B**) compares vehicle to Tolvaptan; (**C**) compares vehicle to JT09 at 5 mg/kg; (**D**) compares vehicle to JT09 at 20 mg/kg; (**E**) compares Tolvaptan to JT09 at 5 mg/kg; (**F**) compares Tolvaptan to JT09 at 20 mg/kg. Analysis was performed with Two-Way ANOVA followed by Dunnett’s post hoc test against saline control (V); Statistical significance: * *p* < 0.05, ** *p* < 0.01, *** *p* < 0.001. Groups: V: saline vehicle, 0.2 mL, PO; T: Tolvaptan, 30 mg/kg, PO; JT09-5mg/kg (J-5): 5 mg/kg, PO; JT09-20mg/kg (J-20): 20 mg/kg, PO. Data are expressed as mean ± standard deviation with n = 5.

The urine analysis revealed a significant increase in urine output (urine volume) in Tolvaptan-treated animals (T) when compared to vehicle control (V), with effects lasting the entire 24-h time period (Figure 2). Similarly, urinalysis of JT09 administered cohorts J-5 and J-20 revealed a significant increase in urine output compared to vehicle control animals. However, urine output in JT09 animals (J-5, J-20) was significantly reduced in comparison to Tolvaptan-treated animals at all time points (Figure 2). The diuretic effects of JT09 lasted for 8 h. No changes in urine appearance or color were noted.

#### 3.2.2. Clinical Chemistry

A significant reduction in serum creatinine was observed at the 5-h time point in Tolvaptan (T), JT09-5mg/kg (J-5), and JT09-20mg/kg (J-20) animals relative to vehicle control (Table A1) (19). In comparison to the vehicle control group (V), serum calcium levels were significantly reduced at 1 h (J-5, J-20), 5 h (T, J-5, J-20), and 24 h (J-5, J-20). In comparison to Tolvaptan, serum calcium levels were significantly reduced at 1 h (J-5, J-20), 5 h (J-20), and 24 h (J-20). Sodium levels did not show consistent elevations or reductions between test groups and controls. Serum sodium significantly increased in Tolvaptan animals at 1 hour; there was no significant increase in sodium levels in J-5 or J-20 animals compared to vehicle control. Sodium levels were significantly reduced in J-5 at 1 h when compared to animals that received Tolvaptan. Serum potassium levels were significantly increased at 1 h (J-5, J-20) compared to vehicle control and Tolvaptan animals. There was a significant reduction in both urea and BUN level observed at 5 h (T, J-5, J-20). In comparison to Tolvaptan, the BUN level was significantly reduced in J-5 animals at 24 h. Compared to vehicle control, glucose levels were significantly increased at 1 h (J-5), 5 h (J-5), and 24 h (J-5, J-20). Further, glucose level was significantly increased in J-20 animals at 24 h when compared to positive control animals. The significant difference in triglyceride, total protein, AST, and chloride at different time points was not consistent throughout the experimental period. All other labs (total cholesterol, albumin, alanine aminotransferase (ALT), alkaline phosphatase (ALP), phosphorus, total bilirubin, and globulin) were statistically indistinguishable from vehicle treatment.

In comparison to vehicle control animals (G1), the urine specific gravity was significantly increased at 2 h in J-5 and J-20 rats (Table 1) [19]; however, urine specific gravity was significantly reduced in J-5 and J-20 rats at 8 h. In comparison to Tolvaptan urine specific gravity was significantly reduced in J-5 rats at 2 h and J-20 rats at 5 h. 

**Figure 3:**
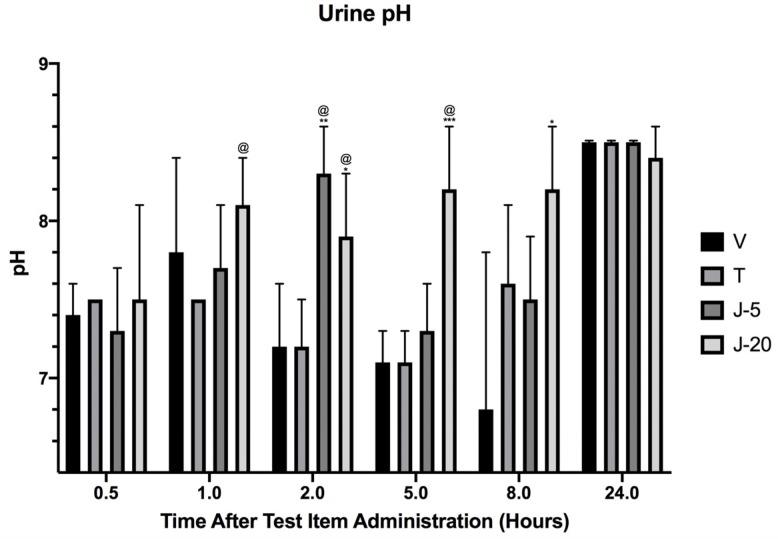
Average urine output measured at 30 min and 1 h, 2 h, 5 h, 8 h and 24 h after single oral or intravenous treatment. Analysis was performed with two-way ANOVA followed by Dunnett’s post hoc test against saline control (V); Statistical significance: * *p* < 0.05, ** *p* < 0.01, *** *p* < 0.001. Groups: V: saline vehicle, 0.2 mL, PO; T: Tolvaptan, 30 mg/kg, PO; JT09-5mg/kg (J-5): 5 mg/kg, PO; JT09-20mg/kg (J-20): 20 mg/kg, PO. Data are expressed as mean ± standard deviation with n = 5.

The urine pH values did now show any significant difference between Tolvaptan and vehicle control rats (Figure 3 [19]). However, a significant increase urine pH was observed in all JT09 administered animals (J-5, J-20). Further, the pH was significantly increased in J-20 animals at 5 and 8 h. In comparison to positive control, the pH value was significantly increased in J-5 and J-20 animals at 2 h and further increased in J-5 animals at 1 h and 5 h. 

## 4. Discussion

This study was performed to assess the diuretic activity of test item, JT09, through single oral administration to Sprague–Dawley rats, relative to vehicle control and Tolvaptan (positive control). Results indicated that JT09 promotes diuresis, but not to the same extent as the positive control. The urinalysis results demonstrated a significant increase in urine output in both Tolvaptan (effects lasting up to 24 h) and all JT09 administered animals (effects lasting up to 8 h), as compared to vehicle control. However, urine output was significantly increased in Tolvaptan rats at all time points relative to all JT09 administered animals. These findings suggest that the diuretic effects of JT09 are not as profound as Tolvaptan; however, JT09 does demonstrate diuretic activity relative to vehicle. This finding establishes an acceptable preliminary diuretic profile for JT09.

In addition to diuresis, alterations in clinical chemistry among JT09 administered rats were observed. Serum biochemistry results revealed a significant reduction in creatinine level in both Tolvaptan and JT09 (J-5, J-20) administered rats. In addition, blood urea and BUN levels were significantly reduced in both Tolvaptan- and JT09-treated animals. Serum creatinine, urea, and BUN levels are commonly measured in patients taking diuretics in order to assess the overall safety profile associated with the therapeutic agent. Furthermore, creatinine, urea, and BUN are commonly used as indicators of kidney function and are subsequently closely monitored. Following administration of a diuretic, plasma volume and serum sodium levels are expected to decrease. This leads to compensatory constriction of the efferent arteriole in order to preserve the glomerular filtration rate (GFR), causing a decrease in renal plasma flow through the glomerulus per unit of time. Thus, it is anticipated that serum creatinine, urea, and BUN levels will increase over time with repeated diuretic use [33,34]. A follow-up study assessing serum chemistry parameters in rats chronically administered JT09 is thus required to draw meaningful conclusions regarding its effects on serum creatinine, urea, and BUN levels. Serum calcium levels were significantly reduced in all JT09 administered animals as compared to vehicle control. However, there was no significant reduction observed in Tolvaptan administered animals. A reduction in serum calcium level is a commonly seen in patients administered thiazide diuretics, such as hydrochlorothiazide [35]. This result is particularly interesting, as it may provide insight into the potential mechanisms by which JT09 promotes diuretic activity. Perhaps the most pertinent finding in this study was that JT09 did not promote a significant increase in serum sodium levels in J-5 and J-20 animals. CR845 was placed on a phase III clinical hold due to the promotion of hypernatremia following single intravenous administration at its highest dose level [36]. Thus, the lack of change in serum sodium levels observed in all JT09 administered rats at high dose levels is a promising preliminary finding. As previously stated, a follow-up study assessing serum chemistry parameters in rats chronically administered JT09 is required. JT09 had an inconsistent effect on serum potassium levels relative to vehicle control. Further, serum potassium levels were significantly increased in J-5 and J-20 animals at the 1 h time point as compared to vehicle control and Tolvaptan treated animals, but were consistent with vehicle control at all other time points. All other labs were normal.

The urine specific gravity was significantly reduced in Tolvaptan administered animals and animals administered JT09 through oral route (J-5, J-20) at 2 and 8 h; however, all urine specific gravity measurements fell within the normal physiological range (1.002–1.030) and are likely a result of the diuretic effects of each compound relative to vehicle control Furthermore, the urine of diuretic treated animals is less concentrated relative to the urine of vehicle control, leading to a significant reduction in urine specific gravity. Additionally, urine pH was increased in all JT09 administered animals (J-5, J-20), except J-20 animals at 5 and 8 h. In comparison to positive control, the pH value was significantly increased in J-5 and J-20 animals at 2 h and further increased in J-20 animals at 1 h and 5 h. The elevation in urine pH may be due to the excretion of JT09, which is a weak base. 

In conclusion, diuretic activity is a desirable effect in patients with cardiovascular disease, hypercalciuria, and advanced liver disease, such as cirrhosis [37]. As previously mentioned, diuretics help relieve the symptoms of congestive heart failure by reducing volume overload and thus, reducing left ventricular pre-load and systemic vascular resistance [3]. Results indicate that JT09 possesses diuretic activity and thus may benefit said patients. In addition to diuretic activity, our laboratory is currently assessing the efficacy of JT09 in rodent models of heart failure (HF) and mitral valve prolapse. As previously stated, KOAs demonstrate the capacity to enhance cardiac performance and reduce cardiac remodeling in the setting of HF. KOAs may also provide synergistic activity when co-administered with other cardiovascular therapeutics, including β-blockers and diuretics [38]. JT09 would be the first orally-available, peripherally-restricted, KOA under development for the treatment of CVD. The side-effect profile associated with JT09 appears to be limited and thus, we believe that JT09 is suitable for development as a broad spectrum therapeutic for the treatment of HF and CVD.

**Limitations of the Study**: There were certain limitations in undertaking this research work. In order to reduce the number of animals used in experimentation, we exclusively used male Sprague–Dawley rats. Despite evidence suggesting a lack of sex-differences in the diuretic activity of selective KOAs, we were unable to assess potential differences among male and female subjects following administration of our lead compound JT09. Additionally, we were unable to use CR845, a selective tetra-peptide KOA with similar chemical properties to JT09, as a positive control. Furthermore, CR845 is currently in phase III clinical trials and was unavailable for purchase as a control. Lastly, the present study assessed the diuretic effects of JT09 following single oral administration. A follow-up study assessing the long-term effects of JT09 on serum chemistry and urine output would provide beneficial insight regarding the safety provide of our lead compound. We believe that the following limitations are out-weighed by the benefits of the study and that the results establish an acceptable preliminary diuretic profile for lead compound JT09.

## Figures and Tables

**Table 1:** Summary of urine specific gravity.

Group and Sex		V & M	T & M	J-5 & M	J-20 & M
Dose (mg/kg/Body Weight)		0	30	5	20
30 min	Mean	1.011	1.011	1.011	1.012
	±SD	0.002	0.002	0.002	0.006
1st h	Mean	1.015	1.011	1.010	1.013
	±SD	0.006	0.002	0.000	0.003
2nd h	Mean	1.021	1.010 *	1.013 *	1.011 *
	±SD	0.004	0.000	0.003	0.002
5th h	Mean	1.021	1.016	1.013	1.018
	±SD	0.006	0.002	0.003	0.004
8th h	Mean	1.021	1.011 *	1.010 *	1.011
	±SD	0.007	0.002	0.000	0.002
24th h	Mean	1.008	1.006	1.007	1.007
	±SD	0.003	0.002	0.003	0.003

Values for urine specific gravity are expressed as Mean ± S.D. (n = 5/group); Analysis was performed with Two-Way ANOVA followed by Dunnett’s post hoc test against saline control (V); Statistical significance: * *p* < 0.05, ** *p* < 0.01, *** *p* < 0.001. Groups: V: saline vehicle, 0.2 mL, PO; T: Tolvaptan, 30 mg/kg, PO; JT09-5mg/kg (J-5): 5 mg/kg, PO; JT09-20mg/kg (J-20): 20 mg/kg, PO. Data are expressed as mean ± standard deviation with n = 5; Abbreviations: G: Group; M: Male; SD: Standard Deviation.

## Appendix A

**Table A1:** Summary of Serum Chemistry Lab.

Time Point	Group, Sex & Dose (mg/kg)		Sodium (mmol/L)	Potassium (mmol/L)	Chloride (mmol/L)	Calcium (mg/dL)	Glucose (mg/dL)	Creatinine (mg/dL)	Blood Urea Nitrogen (mg/dL)	Urea (mg/dL)	Total Protein (g/dL)	Triglycerides (mg/dL)	AST (U/L)
**1st Hour**	**V, M** **& 0**	**Mean**	139.44	5.18	102.56	10.66	126.40	0.48	15.45	33.10	5.86	87.80	214.80
		**±SD**	2.80	0.15	1.59	0.38	11.78	0.02	2.04	11.78	0.17	17.51	51.05
	**T, M** **& 30**	**Mean**	**143.20 ***	5.19	104.16	10.56	132.80	0.50	16.51	35.38	5.86	**100.20 ***	206.20
		**±SD**	**0.78**	0.13	0.84	0.29	10.94	0.02	2.08	4.45	0.30	**28.91**	33.77
	**J-5, M** **& 5**	Mean	139.54	**6.00 ***	101.84	**9.60 ***	**142.80 ***	0.51	14.44	30.94	**6.20 ***	110.60	195.40
		**±SD**	1.82	**0.13**	1.56	**0.36**	**15.43**	0.04	0.98	2.11	**0.17**	24.36	22.79
	**J-20, M** **& 20**	**Mean**	141.24	**5.99 ***	103.26	**9.28 ***	129.60	0.48	16.04	34.38	**6.10 ***	**123.20 ***	202.00
		**±SD**	1.65	**0.25**	1.22	**0.60**	10.31	0.02	1.19	2.56	**0.04**	**17.28**	26.23
**5th Hour**	**V, M** **& 0**	**Mean**	136.78	5.66	100.82	9.72	130.60	0.51	13.23	28.34	5.72	57.80	246.60
		**±SD**	3.17	0.31	2.09	0.23	5.77	0.01	1.18	2.52	0.24	10.33	31.00
	**T, M** **& 30**	**Mean**	137.54	5.27	101.56	**9.36 ***	138.40	0.48	**11.20 ***	**24.00 ***	5.68	82.60	194.40
		**±SD**	1.92	0.56	1.40	**0.21**	9.56	0.03	**0.97**	**2.07**	0.33	40.77	22.93
	**J-5, M** **& 5**	**Mean**	135.82	5.41	100.68	**9.22 ***	136.00	**0.47 ***	**9.79 ***	**20.96 ***	5.92	89.60	**182.60 ***
		**±SD**	1.37	0.33	0.86	**0.31**	3.81	**0.03**	**1.01**	**2.15**	0.16	30.05	**10.01**
	**J-20, M** **& 20**	**Mean**	136.50	5.60	100.90	**8.96 ***	**145.80 ***	**0.46 ***	**10.79 ***	**23.12 ***	5.94	**84.20 ***	254.60
		**±SD**	0.85	0.10	0.52	**0.21**	**7.92**	**0.02**	**1.78**	**3.80**	0.30	**22.41**	125.36
**24th Hour**	**V, M** **& 0**	**Mean**	135.50	5.81	99.24	10.62	143.80	0.54	16.31	34.94	6.18	70.60	203.20
		**±SD**	1.60	0.23	0.24	0.39	3.96	0.03	2.75	5.88	0.42	21.51	31.34
	**T, M** **& 30**	**Mean**	135.18	5.74	99.58	10.52	144.20	0.52	18.49	39.62	5.86	76.20	**164.00 ***
		**±SD**	1.53	0.41	0.72	0.31	12.81	0.05	3.59	7.69	0.29	32.55	**17.76**
	**J-5, M** **& 5**	**Mean**	135.42	5.97	99.72	**10.12 ***	**161.60 ***	0.51	14.06	30.14	6.26	81.40	**155.20 ***
		**±SD**	1.36	0.52	1.08	**0.31**	**8.53**	0.02	2.44	5.23	0.31	11.06	**10.47**
	**J-20, M** **& 20**	**Mean**	135.76	6.21	99.42	**9.92 ***	**170.40 ***	0.51	16.13	34.56	6.16	88.00	206.80
		**±SD**	2.37	0.52	0.97	**0.21**	**19.28**	0.04	0.71	1.51	0.15	19.71	56.47

Values of serum chemistry labs are expressed as Mean ± S.D. (n = 5/group); Analysis was performed with Two-Way ANOVA followed by Dunnett’s post hoc test against saline control (V); Statistically significant values (*p* < 0.05) are identified in bold with an asterisk. Groups: V: saline vehicle, 0.2 mL, PO; T: Tolvaptan, 30 mg/kg, PO; JT09-5mg/kg (J-5): 5 mg/kg, PO; JT09-20mg/kg (J-20): 20 mg/kg, PO. Abbreviations: G: Group; M: Male; SD: Standard Deviation; AST: Aspartate aminotransferase.

### Comments on Table A1

Relative to saline vehicle control (V), Tolvaptan (T) demonstrated statistically significant changes in the following serum chemistry parameters: 1st hour: sodium and triglycerides; 5th hour: Calcium, blood urea nitrogen (BUN), and urea; and 24th hour: aspartate aminotransferase (AST).

Relative to saline vehicle control (V), **JT09** administered orally at 5 mg/kg (J-5) demonstrated statistically significant changes in the following serum chemistry parameters: 1st hour: potassium, calcium, glucose, and total protein; 5th hour: calcium, creatinine, BUN, urea, and AST; and 24th hour: calcium, glucose, and AST.

Relative to saline vehicle control (V), **JT09** administered orally at 20 mg/kg (J-20) demonstrated statistically significant changes in the following serum chemistry parameters: 1st hour: potassium, calcium, total protein, and triglycerides; 5th hour: calcium, glucose, creatinine, BUN, urea, triglycerides; and 24th hour: calcium and glucose.
